# Supplementary material for: Post-Transplant Diabetes Mellitus in Kidney-Transplanted Patients: Related Factors and Impact on Long-Term Outcome
Source: Nutrients. 2024 May 17;16(10):1520. doi: 10.3390/nu16101520 (PMC11123789; doi:10.3390/nu16101520)
Supplement: Supplementary file 1 [file nutrients-16-01520-s001.zip › nutrients-2900606-supplementary.pdf]

Table S1. Division into groups of studied patients.

Groups	Features	Number of Patients
**T**	Total patients	879
**L**	Patients lost at follow up	18
**E**	Patients that didn't reach 6 months of RTX	29
**S**	Patients studied	832
**ND**	NON-diabetic patients	595
**D**	All diabetic patients	237
**D1**	Diabetic patients before transplantation	51
**PTDM+**	Diabetic patients after transplantation	186
**AMG+**	Diabetic and prediabetic patients (IFG or IGT) at OGTT	113 (23+90)
**AMG–**	Patients without OGTT alterations	371

Table S2. Immunosuppressive Therapy; Notes: ATG: Anti-thymocyte globulin: MMF: mycophenolate; * = ND vs. D, ^ = ND vs. PTDM+, ° = AMG+ vs. AMG–. ND: non-diabetic; D: diabetic; PTDM+: post-transplant diabetes mellitus; AMG-: no alterations in glucose metabolsim; AMG+: alterations in glucose metabolism. Non significant *p*-values are reported.

Parameters	T (n = 832)	ND (n = 595)	D (n = 237)	PTDM+ (n = 186)	AMG+ (n = 113)	AMG– (n = 371)	*p*
**Cumulative dose of steroids at T12 (mg)**	2914 ± 962	2923 ± 880	2893 ± 1144	2950 ± 1055	2934 ± 1003	2921 ± 769	*0.696<br>^0.724<br>°0.881
**Basiliximab , %**	77.7%	78.4%	75.8%	81.7%	72.6%	82.5%	*0.459<br>^0.406<br>°0.022
**ATG, %**	24.6%	22.9%	28.8%	22.0%	31.0%	19.9%	*0.089<br>^0.920<br>°0.014
**Ciclosporin/Tacrolimus at T1, %**	7.3%<br>92.7%	7.4%<br>92.6%	7.3%<br>92.7%	8.2%<br>91.8%	5.3%<br>94.7%	4.9%<br>95.1%	*0.348<br>^0.212<br>°0.806
**MMF at T1, %**	94.6%	94.2%	95.8%	96.8%	92.9%	95.7%	*0.398<br>^0.188<br>°0.221
**mTor inhibitors at T1, %**	2.7%	2.6%	3.0%	3.2%	3.5%	2.2%	*0.812<br>^0.609<br>°0.486
**Ciclosporin/Tacrolimus at T12, %**	7.9%<br>92.1%	7.4%<br>92.6%	9.2%<br>90.8%	10.7%<br>89.3%	7.2%<br>92.8%	5.8%<br>94.2%	*0.609<br>^0.371<br>°0.406
**MMF at T12, %**	91.1%	92.3%	88.4%	89.6%	88.3%	94.4%	*0.099<br>^0.281<br>°0.035
**mTor inhibitors at T12, %**	5.8%	4.9%	7.8%	8.2%	7.1%	5.4%	*0.132<br>^0.100<br>°0.489

Table S3. Comparison of clinical and biochemical parameters between ND and D groups; Notes: ND: non-diabetic; D: diabetic; eGFR: estimated glomerular filtration rate BMI: body bass index; SBP: systolic blood pressure; CRP: C-Reactive protein; Only the significant p-values parameters are shown; Significant *p*-values are in bold.

**Parameters**	**1st Month ND**	**1st Month D**	*p*	**6th Month ND**	**6th Month D**	*p*	**12th Month ND**	**12th Month D**	*p*
**eGFR (mL/min)**	66 ± 26	60 ± 26	**0.003**	65 ± 23	57 ± 22	**<0.001**	66 ± 24	59 ± 20	**<0.001**
**Creatinine (mg/dL)**	1.43 ± 0.59	1.50 ± 0.49	0.131	1.41 ± 0.45	2.20 ± 0.59	0.074	1.39 ± 0.46	1.46 ± 0.42	**0.036**
**Proteinuria (g/24h)**	0.20 [0.14–0.30]	0.22 [0.15–0.32]	0.065	0.17 [0.11–0.25]	0.18 [0.12–0.29]	**0.029**	0.16 [0.10–0.25]	0.19 [0.12–0.30]	**0.001**
**BMI (kg/m2)**	22 ± 3	24 ± 3	**<0.001**	-	-	-	23 ± 3	25 ± 3	**<0.001**
**SBP (mmHg)**	130 ± 16	132 ± 16	0.093	130 ± 17	133 ± 18	**0.009**	129 ± 16	132 ± 17	**0.022**
**Glucose (mg/dL)**	82 ± 15	104 ± 31	**<0.001**	85 ± 12	106 ± 34	**<0.001**	82 ± 13	103 ± 34	**<0.001**
**Glycated hemoglobin (mmol/mol)**	33 ± 5	41 ± 8	**<0.001**	36 ± 5	44 ± 9	**<0.001**	35 ± 3	46 ± 9	<0.001
**Total cholesterol (mg/dL)**	210 ± 49	216 ± 56	0.100	197 ± 45	207 ± 45	**0.004**	192 ± 41	201 ± 46	**0.009**
**HDL cholesterol (mg/dL)**	62 ± 19	59 ± 20	0.076	56 ± 19	55 ± 18	0.485	57 ± 18	54 ± 15	**0.020**
**Triglycerides (mg/dL)**	158 ± 74	182 ± 104	**<0.001**	154 ± 75	173 ± 89	**0.002**	142 ± 67	163 ± 68	**<0.001**
**CRP (mg/dL)**	0.14 [0.06–0.48]	0.27 [0.10–0.60]	**0.002**	0.11 [0.06–0.27]	0.18 [0.09–0.33]	**0.008**	0.11 [0.06–0.29]	0.19 [0.09–0.45]	**<0.001**

Table S4. Comparison of clinical and biochemical parameters between ND and D groups; Notes: ND: non-diabetic; D: diabetic; BMI: body bass index; DBP: diastolic blood pressure; PTH: parathormone; Ca: Calcium ; P: Phosphorus ; Mg: Magnesium ; 25-OH VitaminD; 1-25OH(D): 1-25-OH VitaminD. Non significant p-values are reported.

**Parameters**	**1st Month ND**	**1st Month D**	*p*	**6th Month ND**	**6th Month -D**	*p*	**12th Month ND**	**12th Month D**	*p*
**DBP (mmHg)**	79 ± 10	79 ± 10	0.618	80 ± 10	79 ± 10	0.310	79 ± 10	79 ± 9	0.411
**Uric acid (mg/dL)**	5.78 ± 1.48	5.82 ± 1.82	0.760	6.49 ± 1.48	6.55 ± 1.62	0.593	6.50 ± 1.50	6.65 ± 1.66	0.216
**Hemoglobin (g/dL)**	11.0 ± 1.36	10.89 ± 1.36	0.233	12.32 ± 1.53	12.22 ± 1.36	0.354	12.81 ± 1.66	12.65 ± 1.45	0.180
**Albumin (g/dL)**	4.15 ± 0.43	4.11 ± 0.42	0.220	4.42 ± 0.36	4.36 ± 0.37	0.061	4.42 ± 0.34	4.36 ± 0.40	0.064
**PTH (pg/mL)**	64 [39–103]	61 [37–107]	0.535	56 [39–91]	61 [37–98]	0.710	56 [38–89]	54 [35–83]	0.261
**Ca (mg/dL)**	9.76 ± 0.80	9.67 ± 0.76	0.132	9.86 ± 0.76	9.81 ± 0.62	0.357	9.83 ± 0.74	9.80 ± 0.58	0.666
**P (mg/dL)**	2.55 ± 0.88	2.53 ± 0.96	0.769	3.09 ± 0.71	3.12 ± 0.68	0.550	3.12 ± 0.65	3.17 ± 0.61	0.308
**Mg (mg/dL)**	1.63 ± 0.23	1.62 ± 0.21	0.548	1.74 ± 0.21	1.73 ± 0.24	0.862	1.70 ± 0.21	1.74 ± 0.30	0.230
**Insulinemia (μIU/mL)**	8.50 [6.20–11.70]	9.90 [6.30–13.05]	0.077	8.20 [5.80–11.55]	8.17 [6.00–12.20]	0.343	8.60 [6.00–11.30]	8.70 [5.90–11.90]	0.893
**Alkaline phosphatase (U/L)**	87 [67–117]	90 [69–127]	0.174	88 [66–125]	95 [71–123]	0.076	81 [60–109]	88 [66–113]	**0.037**
**25OH(D) (ng/dL)**	14.54 ± 7.91	14.17 ± 7.99	0.592	16.65 ± 9.65	16.84 ± 10.95	0.839	19.15 ± 10.73	19.45 ± 13.32	0.762
**1-25OH(D) (ng/dL)**	38.39 ± 24.02	38.18 ± 23.49	0.936	49.69 ± 21.14	47.66 ± 20.07	0.369	53.11 ± 21.54	50.97 ± 19.16	0.343

Table S5. Comparison of clinical and biochemical parameters between the ND and PTDM+ groups. ND: non-diabetic; PTDM+: post-transplant diabetes mellitus; eGFR: estimated glomerular filtration rate BMI: body bass index; SBP: systolic blood pressure; CRP: C-Reactive protein; Only the significant *p*-values parameters are shown; Significant *p*-values are in bold.

Parameters	1st Month ND	1st Month PTDM+	*p*	6th Month ND	6th Month PTDM+	*p*	12th Month ND	12th Month PTDM+	*p*
**eGFR (mL/min)**	66 ± 26	60 ± 26	**0.012**	65 ± 23	57 ± 2255	**<0.001**	66 ± 24	59 ± 20	**0.001**
**Creatinine (mg/dL)**	1.43 ± 0.59	1.48 ± 0.49	0.150	1.41 ± 0.45	2.39 ± 11.96	**0.050**	1.39 ± 0.46	1.47 ± 0.42	**0.042**
**Proteinuria (g/24h)**	0.20 [0.14–0.30]	0.21 [0.15–0.31]	0.176	0.17 [0.11–0.25]	0.18 [0.12–0.28]	0.218	0.16 [0.10–0.25]	0.18 [0.12–0.30]	**0.006**
**BMI (kg/m2)**	22 ± 3	24 ± 3	**<0.001**	-	-	-	23 ± 3	25 ± 3	**<0.001**
**SBP (mmHg)**	130 ± 16	132 ± 16	0.084	130 ± 17	134 ± 18	**0.006**	129 ± 16	132 ± 17	**0.013**
**Blood glucose (mg/dL)**	82 ± 15	100 ± 29	**<0.001**	85 ± 12	100 ± 26	**<0.001**	82 ± 13	96 ± 23	**<0.001**
**Insulinemia (μIU/mL)**	8.50 [6.20–11.70]	9.90 [7.00–13.00]	**0.035**	8.20 [5.80–11.55]	8.25 [6.00–12.35]	0.176	8.60 [6.00–11.30]	8.80 [5.90–11.90]	0.728
**Glycated hemoglobin (mmol/mol)**	33 ± 5	41 ± 8	**<0.001**	36 ± 5	44 ± 8	**<0.001**	35 ± 3	45 ± 7	**<0.001**
**Total cholesterol (mg/dL)**	210 ± 49	225 ± 51	**<0.001**	197 ± 45	213 ± 45	**<0.001**	192 ± 41	206 ± 45	**<0.001**
**Triglycerides (mg/dL)**	158 ± 74	187 ± 109	**<0.001**	154 ± 75	177 ± 92	**0.001**	142 ± 67	166 ± 71	**<0.001**
**CRP (mg/dL)**	0.14 [0.06–0.48]	0.29 [0.10–0.60]	**0.002**	0.11 [0.06–0.27]	0.20 [0.09–0.34]	**0.002**	0.11 [0.06–0.29]	0.20 [0.09–0.46]	**<0.001**

Table S6. Comparison of clinical and biochemical parameters between the ND and PTDM+ groups; Notes: ND: non-diabetic; PTDM+: post-transplant diabetes mellitus; DBP: diastolic blood pressure; PTH: parathormone; Ca: Calcium ; P: Phosphorus ; Mg: Magnesium ; 25-OH VitaminD; 1-25OH(D): 1-25-OH VitaminD. Not significant p-values are reported.

Parameters	1st Month ND	1st Month PTDM+	*p*	6th Month ND	6th Month PTDM+	*p*	12th Month ND	12th Month PTDM+	*p*
**DBP (mmHg)**	79 ± 10	79 ± 9	0.899	80 ± 10	80 ± 9	0.801	79 ± 10	80 ± 9	0.867
**Uric acid (mg/dL)**	5.78 ± 1.48	5.91 ± 1.84	0.373	6.49 ± 1.48	6.62 ± 1.60	0.268	6.50 ± 1.50	6.75 ± 1.58	0.050
**Hemoglobin (g/dL)**	11.01 ± 1.36	10.92 ± 1.36	0.401	12.32 ± 1.53	12.26 ± 1.39	0.612	12.81 ± 1.66	12.65 ± 1.49	0.229
**Albumin (g/dL)**	4.15 ± 0.43	4.15 ± 0.38	0.959	4.42 ± 0.36	4.39 ± 0.36	0.382	4.42 ± 0.34	4.40 ± 0.39	0.594
**PTH (pg/mL)**	64 [39–103]	62 [38–107]	0.613	56 [39–91]	61 [37–95]	0.793	56 [38–89]	55 [35–80]	0.331
**Ca (mg/dL)**	9.76 ± 0.80	9.69 ± 0.79	0.270	9.86 ± 0.76	9.81 ± 0.62	0.468	9.83 ± 0.74	9.80 ± 0.57	0.694
**P (mg/dL)**	2.55 ± 0.88	2.55 ± 0.99	0.970	3.09 ± 0.71	3.13 ± 0.69	0.514	3.12 ± 0.65	3.20 ± 0.61	0.144
**Mg (mg/dL)**	1.63 ± 0.23	1.61 ± 0.19	0.457	1.74 ± 0.21	1.77 ± 0.23	0.358	1.70 ± 0.21	1.73 ± 0.34	0.492
**Alkaline phosphatase (U/L)**	87 [67–117]	90 [69–124]	0.352	88 [66–125]	94 [71–122]	0.106	81 [60–109]	88 [67–112]	0.077
**HDL cholesterol (mg/dL)**	62 ± 19	60 ± 21	0.330	56 ± 19	55 ± 19	0.660	57 ± 18	54 ± 16	0.082
**25OH(D) (ng/dL)**	14.54 ± 7.91	13.77 ± 7.26	0.307	16.65 ± 9.65	16.83 ± 11.26	0.858	19.15 ± 10.73	19.58 ± 13.75	0.708
**1-25OH(D) (ng/dL)**	38.39 ± 24.02	38.88 ± 25.94	0.873	49.69 ± 21.14	49.15 ± 19.95	0.857	53.11 ± 21.54	52.15 ± 19.90	0.724

Table S7. Comparison of clinical and biochemical parameters between the AMG– and AMG+ groups; Notes: AMG-: no alterations in glucose metabolsim; AMG+: alterations in glucose metabolism; eGFR: estimated glomerular filtration rate BMI: body bass index; SBP: systolic blood pressure; DBP: diastolic blood pressure; PTH: parathormone; CRP: C-Reactive protein; 25-OH VitaminD. Significant *p*-values are in bold.

Parameters	1st Month AMG–	1st Month AMG+	*p*	6th Month AMG–	6th Month AMG+	*p*	12th Month AMG–	12th Month AMG+	*p*
**eGFR (mL/min)**	67 ± 25	59 ± 22	**0.004**	65 ± 23	58 ± 23	**0.002**	67 ± 23	60 ± 26	**0.004**
**Creatinine (mg/dL)**	1.38 ± 0.46	1.51 ± 0.59	**0.015**	1.82 ± 8.45	1.54 ± 0.51	0.725	1.34 ± 0.38	1.49 ± 0.54	**0.001**
**Proteinuria (g/24h)**	0.20 [0.14–0.30]	0.21 [0.14–0.33]	0.215	0.17 [0.11–0.24]	0.18 [0.12–0.28]	0.067	0.17 [0.10–0.25]	0.19 [0.12–0.26]	**0.045**
**BMI (kg/m2)**	22.88 ± 3.66	24.25 ± 3.41	**0.001**	-	-	-	23.87 ± 3.74	24.91 ± 3.23	**0.009**
**SBP (mmHg)**	129 ± 16	131 ± 16	0.216	129 ± 16	132 ± 14	0.101	127 ± 15	133 ± 17	**0.002**
**DBP (mmHg)**	79 ± 10	79 ± 9	0.900	79 ± 10	79 ± 9	0.848	79 ± 10	81 ± 9	**0.023**
**Uric acid (mg/dL)**	5.70 ± 1.54	5.88 ± 1.60	0.300	6.35 ± 1.43	6.52 ± 1.64	0.313	6.33 ± 1.36	6.66 ± 1.55	**0.031**
**PTH (pg/mL)**	57 [36–100]	68 [45–121]	0.055	51 [37–86]	68 [40–106]	**0.020**	51 [35–81]	68 [40–106]	**0.011**
**Blood glucose (mg/dL)**	80 ± 13	92 ± 21	**<0.001**	85 ± 11	99 ± 12	**<0.001**	81 ± 11	94 ± 16	**<0.001**
**Glycated hemoglobin (mmol/mol)**	34 ± 5	37 ± 6	**<0.001**	37 ± 5	41 ± 6	**<0.001**	37 ± 4	41 ± 6	**<0.001**
**HDL cholesterol (mg/dL)**	62 ± 18	60 ± 22	0.476	56 ± 16	52 ± 14	**0.051**	57 ± 17	53 ± 14	**0.024**
**Triglycerides (mg/dL)**	160 ± 75	163 ± 73	0.640	151 ± 67	179 ± 94	**0.001**	138 ± 61	158 ± 73	**0.006**
**CRP (mg/dL)**	0.14 [0.06–0.40]	0.25 [0.09–0.74]	**0.032**	0.10 [0.05–0.26]	0.16 [0.10–0.40]	**<0.001**	0.11 [0.06–0.30]	0.19 [0.10–0.40]	**<0.001**
**25OH(D) (ng/dL)**	14.96 ± 7.74	12.85 ± 6.49	**0.022**	16.64 ± 9.59	16.54 ± 11.20	0.930	19.22 ± 10.99	18.23 ± 11.72	0.461

Table S8. Comparison of clinical and biochemical parameters between the AMG– and AMG+ groups; Notes: AMG-: no alterations in glucose metabolsim; AMG+: alterations in glucose metabolism; Ca: Calcium ; P: Phosphorus ; Mg: Magnesium ; 1-25OH(D): 1-25-OH VitaminD. Not significant p-values are reported.

Parameters	1st Month AMG–	1st Month AMG+	*p*	6th Month AMG–	6th Month AMG+	*p*	12th Month AMG–	12th Month AMG+	*p*
**Hemoglobin (g/dL)**	11.01 ± 1.36	10.99 ± 1.26	0.871	12.31 ± 1.45	12.35 ± 1.26	0.778	12.79 ± 1.55	12.62 ± 1.42	0.323
**Albumin (g/dL)**	4.20 ± 0.40	4.16 ± 0.41	0.350	4.45 ± 0.34	4.41 ± 0.39	0.264	4.43 ± 0.34	4.39 ± 0.33	0.316
**Ca (mg/dL)**	9.76 ± 0.78	9.68 ± 0.80	0.343	9.86 ± 0.69	9.86 ± 0.68	0.994	9.83 ± 0.72	9.78 ± 0.63	0.548
**P (mg/dL)**	2.58 ± 0.83	2.45 ± 0.85	0.143	3.10 ± 0.64	3.06 ± 0.75	0.564	3.14 ± 0.64	3.09 ± 0.57	0.432
**Mg (mg/dL)**	1.62 ± 0.25	1.63 ± 0.21	0.817	1.73 ± 0.19	1.79 ± 0.27	0.170	1.70 ± 0.22	1.71 ± 0.29	0.868
**Insulinemia (µIU/mL)**	8.60 [6.35–11.63]	10.00 [6.99–13.65]	0.169	8.10 [5.90–11.48]	9.25 [6.43–13.23]	0.053	8.60 [5.90–10.90]	7.60 [5.90–10.00]	0.360
**Alkaline phosphatase (U/L)**	84 [67–112]	90 [70–129]	0.059	88 [65–119]	87 [66–122]	0.479	83 [60–105]	79 [65–116]	0.208
**Total cholesterol (mg/dL)**	214 ± 47	212 ± 51	0.709	199 ± 43	207 ± 49	0.078	193 ± 38	200 ± 41	0.159
**1-25OH(D) (ng/dL)**	41.05 ± 24.4	41.60 ± 28.01	0.894	51.55 ± 21.31	50.51 ± 21.92	0.747	55.76 ± 21.04	49.28 ± 21.72	0.053
